# Hybrid-Capture Target Enrichment in Human Pathogens: Identification, Evolution, Biosurveillance, and Genomic Epidemiology

**DOI:** 10.3390/pathogens13040275

**Published:** 2024-03-23

**Authors:** Z. B. Randolph Quek, Sock Hoon Ng

**Affiliations:** Defence Medical & Environmental Research Institute, DSO National Laboratories, Singapore 117510, Singapore

**Keywords:** neglected tropical diseases, zoonoses, oncoviruses, clinical metagenomics, bait design

## Abstract

High-throughput sequencing (HTS) has revolutionised the field of pathogen genomics, enabling the direct recovery of pathogen genomes from clinical and environmental samples. However, pathogen nucleic acids are often overwhelmed by those of the host, requiring deep metagenomic sequencing to recover sufficient sequences for downstream analyses (e.g., identification and genome characterisation). To circumvent this, hybrid-capture target enrichment (HC) is able to enrich pathogen nucleic acids across multiple scales of divergences and taxa, depending on the panel used. In this review, we outline the applications of HC in human pathogens—bacteria, fungi, parasites and viruses—including identification, genomic epidemiology, antimicrobial resistance genotyping, and evolution. Importantly, we explored the applicability of HC to clinical metagenomics, which ultimately requires more work before it is a reliable and accurate tool for clinical diagnosis. Relatedly, the utility of HC was exemplified by COVID-19, which was used as a case study to illustrate the maturity of HC for recovering pathogen sequences. As we unravel the origins of COVID-19, zoonoses remain more relevant than ever. Therefore, the role of HC in biosurveillance studies is also highlighted in this review, which is critical in preparing us for the next pandemic. We also found that while HC is a popular tool to study viruses, it remains underutilised in parasites and fungi and, to a lesser extent, bacteria. Finally, weevaluated the future of HC with respect to bait design in the eukaryotic groups and the prospect of combining HC with long-read HTS.

## 1. Introduction 

In severe clinical infections, time is of the essence, and rapid identification is critical for patient well-being and formulation of an appropriate treatment plan. Currently, techniques such as qPCR, fluorescent in situ hybridization, DNA microarray hybridization, and different sequencing strategies (marker genes, metagenomics, and whole-genome sequencing) are adopted to verify and identify pathogens [1,2]. With the advent of the molecular revolution, high-throughput sequencing (HTS) transformed the landscape of pathogen genomics. Indeed, researchers can formulate various sequencing strategies depending on the targeted outcome, such as investigating pathogen biology, evolution, or identification.

Pathogens can be identified from clinical and environmental samples using metagenomic sequencing—deep sequencing to recover pathogen genomic sequences [3,4]. The information generated from rapid pathogen identification is critical for guiding rapid response policies [4,5,6,7]. For example, the severe acute respiratory syndrome coronavirus 2 (SARS-CoV-2) was first characterised using metagenomic sequencing when unknown cases of pneumonia began appearing in Wuhan, China (see Section 4. Case Study: SARS-CoV-2). Nevertheless, recovery of pathogen sequences can be challenging due to the overwhelming background sequences from the host [8,9]. To circumvent this, enrichment techniques such as PCR or hybrid-capture target enrichment (subsequently abbreviated as HC) can be used to recover sequences of interest. An example of the former comprises broad-range primers in PCR amplification for specific markers such as 16S rRNA in bacteria [10] or an internal transcribed spacer in fungi [11]. However, marker-based techniques are limited by primer specificity, target coverage, length, and variability of the amplification product [12]. Furthermore, the polyphyletic origin of viruses precludes conserved genes across the viral branch, making it impossible for universal markers to be designed as in the case of bacteria, fungi, or parasites [13]. 

As an alternative enrichment technique, HC alleviates some problems encountered in metagenomic and amplicon-based sequencing. This method involves specific biotinylated DNA or RNA baits (probes) that hybridise to regions of interest (e.g., coding regions and ultra-conserved elements). Following the capture process of target sequences, the unhybridised sequences are washed off, and the remaining fragments are subsequently enriched. In contrast to PCR with shorter oligonucleotide primers, a HC panel typically comprise hundreds to thousands of baits (or probes), ranging from 75 nt to 140 nt in length, designed based on target references (e.g., genomes and transcriptomes). The flexibility of bait design enables a broader range of regions/taxa to be targeted, and the longer oligonucleotides reduce binding bias. Ultimately, HC streamlines processes and reduces noise, leading to more efficient and rapid analyses. Indeed, the versatility of HC has resulted in a myriad of applications, including but not limited to the following reviews for phylogenomics [14], pathogen identification [12,15], paleomicrobiology [16,17], and oncology [18], among others. For example, in one remarkable application of HC, researchers were able to improve the recovery of ancient human DNA from 1.2% to 59% mapped reads and subsequently improve the resolution of population genomic analyses [19]. In that vein of paleobiology, the study of ancient human pathogen genomes is useful in helping our understanding regarding the evolutionary dynamics of infectious diseases. From samples ranging between 2500 and 5000 years old, Valtueña et al. [20] were able to trace the spread of the bubonic plague (*Yersinia pestis*). They found that the spread closely mirrored patterns of human movement, providing clues on the epidemiology of the disease and improving our understanding of *Y. pestis* evolution. Nevertheless, PCR outperforms HC in several aspects, such as (1) cost: design and manufacture of baits are significantly more expensive compared to primers; and (2) time: PCR amplification requires several hours only, in contrast to HC, which may span several days.

Recently, Gaudin and Desnues [15] reviewed advancements in HC with respect to human infectious diseases, and Pei et al. [12] more broadly covered targeted sequencing (amplicon/HC) in clinical settings. However, both reviews were limited in coverage, with the former being a brief review and the latter focused on two distinct targeted sequencing methods. We refer readers interested in comparing PCR and HC target enrichment methods to both reviews. Instead, this review exclusively discusses the application of HC methods applied to pathogens of interest to humans and provides a comprehensive discussion on biosurveillance and emerging infectious diseases, a field especially pertinent after the recent pandemic. Furthermore, we evaluated the applications and future of HC. Our search strategy was as follows: on Google Scholar, we limited studies to between 2011 and March 2023, with the former year selected based on the earliest study using HC covered in Gaudin and Desnues [15]. Keywords “hybrid-capture target enrichment” was used in all search attempts, in conjunction with “human pathogen” and “human infectious disease” tags, up to the first 10 pages of search results (as of February 2023). Other studies the authors encountered were also duly included. Finally, to demonstrate the contemporary popularity and applicability of HC to novel pathogens, we selected SARS-CoV-2 as a case study. A similar search strategy was adopted, with the addition of the keyword “SARS-CoV-2”.

## 2. Hybrid-capture target enrichment for Pathogens

### 2.1. Bacteria

#### 2.1.1. Hybrid Capture versus Amplicon Sequencing in 16S rRNA

Originating billions of years ago with at a conservative estimate of several million species [21], it is difficult to design a bait set that is able to comprehensively sample across all bacteria taxa. One approach to this would be to design baits based on conserved genes, such as the 16S rRNA—a marker commonly used in bacteria classification and identification. Traditionally, the 16S rRNA characterization approach was based on the principle that there were nine hypervariable regions across the gene, and specific, universal primers were designed to amplify regions of interest for sequencing [22]. However, this approach suffers from drawbacks such as primer binding biases and short amplicons, which make it difficult to identify accurate species [23]. With the advent of long-read sequencing, full-length 16S sequences can be directly recovered from clinical samples, which confers similar benefits to HC methods (i.e., longer contiguous sequence recovered) [24]. Importantly, 16S rRNA HC overcomes limitations associated with 16S amplicon sequencing by reducing primer binding biases, providing better relative abundance and community composition estimates, and having unparalleled versatility based on a targeted taxonomic group for bait design [25,26]. While 16S rRNA HC in both studies yielded promising results despite using only a relatively small number of baits (<40,000), 16S rRNA approaches alone are limited in two aspects: (1) accurately classifying sequences to species due to low sequence variability, intragenomic variation between species in some genera, and (2) lack of information on virulence and antibiotic resistance genes [12,27,28]. Therefore, the applications of 16S rRNA HC alone might be better used in community analyses for metagenomic studies and preliminary identification of taxa rather than specific pathogen identification.

#### 2.1.2. Antimicrobial Resistance

Treatment of bacterial infections is inextricably linked to antimicrobial resistance (AMR). In 2019 alone, an estimated 4.95 million deaths were attributed partially to bacterial AMR [29]. Concerningly, the evolution of AMR outpaces the rate of antibiotic discovery, exacerbating the global crisis of AMR pathogen infections [30]. Information on AMR markers facilitates judicious use of antibiotics, monitoring and surveillance efforts of drug-resistant taxa, and sheds light on AMR mechanisms [31,32]. Researchers have designed HC panels solely targeting AMR genes [33,34,35,36] or in conjunction with pathogen identification [37]. Using both cultured multidrug-resistant bacteria and human stool samples, Guitor et al.’s [35] AMR-specific panel was able to increase sequence recovery by several fold, which performed better than earlier panels with a higher percentage (average = 50.69%) of on-target reads (median = 15.8% in [33]; average = 30.26% in [34]). Similarly, Ferreira et al.’s [36] panel of baits for AMR markers outclassed metagenomic sequencing, increasing from 1.5% to 61% on-target reads. Recently, researchers combined long-read sequencing with HC (target-enriched long-read sequencing: TELSeq) to characterise AMR genes from metagenomic samples (human faeces, cow faeces, and soil) [38], using the panel designed in [33]. With the long, contiguous sequences recovered, they were able to further our understanding of AMR evolution by identifying a number of AMR genes proximal to mobile genetic elements, which increases the propensity for transfer of AMR genes between pathogens via horizontal gene transfer (see also [39]). Understanding the evolutionary dynamics of AMR may help researchers combat the growing crisis of antibiotic resistance and guide treatment in the future [40].

#### 2.1.3. Genome Characterisation of Fastidious Bacteria Using Hybrid Capture

Metagenomics and HC can be applied to fastidious or difficult-to-culture bacteria for genome assembly from clinical or environmental samples and subsequent downstream applications (e.g., vaccine development), bypassing the need for culture. *Treponema pallidum*, the causative agent of syphilis, was impervious to long-term in vitro cultivation until recent breakthroughs [41]. To recover *T. pallidum* genomes directly from clinical samples, Pinto et al. [42] developed 19,094 baits covering ~1.1 Mb of six publicly available genomes. Interestingly, they found that patient-derived samples shared a single trait unique to clinical strains and not reference strains: a single nucleotide polymorphism in *mrcA*, which potentially codes for a penicillin-binding protein, thereby decreasing their susceptibility to certain classes of antibiotics. Two other fastidious pathogens of interest, *Bacillus anthracis* and *Mycoplasma amphoriforme*, were targeted using baits designed using only core chromosomal genomes as references, excluding accessory nucleic materials [43]. DNA extracts from animal carcasses and nasopharyngeal swabs/aspirates from clinical samples were used for *B. anthracis* and *M. amphoriforme,* respectively, for empirical validation. Post-enrichment, a sizable fraction of the genomes in breadth and depth were recovered, enabling robust genotyping. In the clinical setting, fastidious infectious agents cannot be cultured readily, but HC can help enrich the pathogen sequences for downstream genomic analyses (e.g., genotyping, virulence, and AMR).

#### 2.1.4. Broad Spectrum Bacteria Bait Panels 

In an attempt to produce an almost universal bait set for capturing pathogenic bacteria sequences, Allicock et al. [37] used the coding sequences of 307 species of pathogenic bacteria in humans, along with known antimicrobial resistance genes and virulence factors (*n* = 1,007,426 genes post-clustering at 96%) for bait design. Due to the scale of the design, over 4 million oligonucleotide baits (average length = 75 nt) were designed, a set that would be astronomically expensive to produce. The ambitious panel designed was targeted at providing a potential diagnosis for bacterial pathogens for which standard tests are unable to provide a resolution, and despite the taxonomic breadth targeted, tests on mock communities demonstrate an enrichment efficacy of up to 1000-fold. In an application of BacCapSeq to clinical samples, researchers enriched bacteria nucleic acids to determine pathogens responsible for neuroinfectious diseases in 34 patients from New York [44]. Importantly, while they found bacteria pathogens in four out of six patients, none of the pathogens were suspected to be the causative agent by clinicians based on the symptoms presented, and some species recovered could be commensals instead (e.g., *Herbaspirillum*). Therefore, the authors advised prudence when interpreting the accuracy of HTS data, which are best used in conjunction with clinical evidence.

More recently, an algorithm for more efficient bait design based on clustering conserved gene families in an evolutionary framework was introduced and implemented in HUBDesign [45]. This economical approach resulted in only 26,870 baits, covering 2.09% of all nucleotides at an average depth of coverage of 3.64× in sepsis bacterial pathogens (1926 bacteria genomes, 81 species). For comparison, the authors postulated that some 2 million baits would be required to cover 2% at a depth of 5×, given a naïve approach to bait design. Designing suitable baits spanning deep evolutionary time is challenging, but new advancements in bait design and sequencing platforms will likely result in a variety of panels. While clinical applications of HC in bacteria pathogen identification remain limited, improvements in algorithms and the usefulness of HC will likely result in more panels being made available in the future. Eventually, with more clinical testing and verification to refine and improve techniques, HC can complement or potentially provide alternative clinical diagnoses. While HTS metagenomics remains a promising avenue for pathogen identification, which can be enhanced using HC, it remains an auxiliary tool for clinicians to tap into (see [46]). 

### 2.2. Viruses

**Figure 1:**
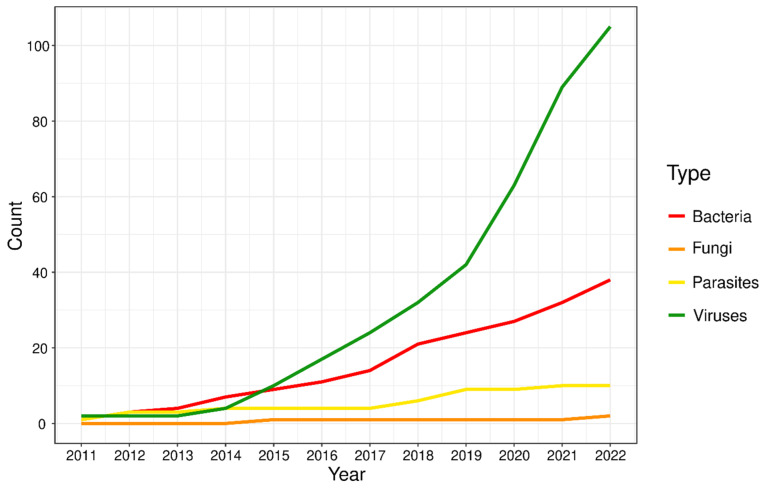
Cumulative number of studies using hybrid-capture target enrichment for human pathogens between 2011 and 2022.

Viruses are popular targets for HC, with a sizable increase in the number of studies in recent years (Figure 1, Table S1). Indeed, the number of studies employing HC in viruses more than doubles that of the closest contender (bacteria), albeit the spike since 2020 is partly due to HC studies in SARS-CoV-2. Furthermore, beyond pathogen identification and transcriptome characterisation, the potential of HC in human pathogen biology is only limited by the imagination of the researcher. For example, a recent study extended the application by combining spatial transcriptomics of both host (human) and pathogen (SARS-CoV-2) from formalin-fixed, paraffin-embedded (FFPE) samples [47]. The colocalization analyses were able to determine differential gene expression of genes, such as those involved in the immune response by the host and entry factors by the pathogen. The success of this experiment paved the way for future investigations in spatial transcriptomics for different pathogens, guiding us to a better understanding of host–pathogen interactions.

#### 2.2.1. Oncoviruses

Oncogenic viruses are among the most popular targets for HC, for good reason. A recent study estimated that the projected cost of cancer between 2020 and 2050 stood at over USD 25 trillion dollars [48], with over 12% of cases attributed to just seven oncogenic viruses: Merkel cell polyomavirus (MCPyV), Epstein–Barr virus (EBV), hepatitis B (HBV) and C (HCV) viruses, human papillomaviruses (HPV), Kaposi’s sarcoma herpesvirus (HHV-8), and human T-cell lymphotropic virus 1 (HTLV-1) [49]. Interestingly, all of the viruses have been investigated using HC techniques: understanding the transmission chain and mechanisms of viral oncogenesis is critical for developing therapeutic strategies for cancer [50,51,52].

In an early experiment targeting viruses using HC, Duncavage et al. [53] created in-house biotinylated baits derived from PCR amplification across the genome of MCPyV for HC. From FFPE samples with degraded DNA, they were able to enrich for viral nucleic acids between 28,000- and 107,000-fold, gathering clues into MCPyV insertion sites and implications on Merkel cell carcinoma. In the same year, Depledge et al. [54] took a more conventional approach, designing baits for three herpesviruses: Varicella–Zoster (VZV), EBV, and HHV-8, of which the latter two have been linked to oncogenesis [55]. Full genome consensus sequences across multiple viruses were generated from a single clinical sample, suggesting co-infections. The same panel was also used to determine the genetic diversity of EBV from primary nasopharyngeal carcinoma (NPC) biopsy samples using HC [56], based on the assembly of eight new EBV genomes. Relatedly, Xu et al. [57] used a commercial kit (MyGenostics Virus Genome Capture System) on tumour samples to identify breakpoints at which EBV integrates into tumour cells, elucidating tumorigenesis in EBV-associated cancers. The recovery and assembly of pathogen genomes from clinical samples can contribute to future studies developing therapeutics for EBV-related cancers [58,59,60]. 

There is a growing body of evidence linking human cytomegaloviruses (HCMV) to oncogenesis, and genomics will be a powerful avenue for future endeavours in treatment and therapeutic approaches [61,62,63]. Immunocompromised hosts are more susceptible to HCMV, making it an important pathogen for clinicians to consider [64]. Reconstructing the phylogeny and population structure of HCMV recovered from patients across different demographics using a custom panel of baits (15 whole and 44 partial HCMV genomes), researchers in London found high recombination rates across the genome, except at loci under positive selection related to immune evasion [65], which could potentially explain the high prevalence of HCMV [66,67]. Furthermore, a separate HC study found that the dominant HCMV strain was observed to switch in individuals with multiple-strain infections during their lifetime, which might be implicated in HCMV pathogenesis, and single-strain infections were significantly more common in congenitally infected patients as compared to transplant recipients [68,69]. Using the same panel, analyses of breast milk from women with HIV women found multiple HCMV strains [70], corroborating previous evidence that the establishment of HCMV infection is likely limited by strain in transmission between mother and child [71,72].

Hepatitis is a deadly disease, and in chronic cases, HBV and HCV have been linked to cancers such as non-Hodgkin lymphoma and liver cancer [73,74]. While treatments are available, albeit being underdiagnosed and untreated [75], drug-resistance hampers treatment, which may be elucidated via genotyping. In a panel designed for HCV HC (titled ve-SEQ), baits were added incrementally, referencing 482 genomes to baits from an initial 4-genotype panel [76]. Genotyping of 29 clinical samples revealed possible drug resistance in some strains detected, which corresponded with reduced drug efficacy: Boceprevir and Telaprevir treatment did not suppress HCV due to mutations in the NS3 gene in two patients. Usually, the detection of resistance-associated variants (RAVs) in HCV is identified using PCR methods, but divergent genotypes and mixed infections may mask accurate characterisation, which can be easily bypassed using HC [77]. Indeed, by designing variable baits, HC is able to circumvent problems associated with primer specificity given the high genetic diversity in HCV and genotype drug resistance mutations, which optimises treatment and reduces costs from futile drug prescription, making it an invaluable tool for clinicians [77,78]. Interestingly, a recent study leveraged HC to recover HBV reads from Ludwig van Beethoven’s hair samples, which possibly caused liver disease in the famed composer [79]. Post-HC, the authors were able to recover slightly less than 100 unique HBV reads (compared to four from metagenomic sequencing only) from the hair of Beethoven, which was sufficient to establish the lineage of HBV infected to be nested within the HBV subgenotype D2. 

Human papillomaviruses can be classified as either low-risk, manifesting as genital warts, or high-risk types, potentially resulting in HPV-associated oncogenesis [80,81]. Using 191 HPV reference genomes representing five genera, 23,941 baits (120 nt) were used to capture HPV nucleic acids from laboratory cell lines, achieving an average of 184,483-fold enrichment [82]. In a follow-up study, the reproducibility and sensitivity of their assay were tested with known copy numbers of HPV types in two cell lines: SiHa and HeLa [83]. Not only does the HC method have comparable sensitivity to PCR-based methods, but biases introduced via consensus primers in multi-type infections were not observed, and no significant enrichment was observed for any HPV type tested. With more than 400 types of HPV catalogued [84], accurate typing enables researchers to better understand the risks of HPV infections, develop and update vaccines when necessary, based on longitudinal and/or cohort studies [85,86], and fine-scale resolution can be achieved using HC.

#### 2.2.2. Human Immunodeficiency Virus

The human immunodeficiency virus (HIV) infects millions around the world; in 2019 alone, almost 700,000 fatalities were due to HIV-related illness [87]. Furthermore, HIV takes the pole position in terms of funding—from 2000 to 2017, about 40% (USD 42.1 billion) of funding for research into infectious diseases by G20 countries was allocated to HIV/AIDS alone [88]. Correspondingly, we found a number of HC studies pertaining to HIV research alone, in contrast to other viruses (e.g., HCMV and HHV-8) (Table S1). 

Currently, reducing HIV mortality has made giant strides since the implementation of antiretroviral therapy, but the elimination of the integrated virus in its latent stage remains elusive [89]. To investigate HIV integration sites in host cells, a small panel comprising 52 baits to capture a 72 bp region in the U3 region was designed across 573 long terminal repeat sequences post-clustering (500-fold enrichment) [90]. When tested on cell models (ACH-2, J-Lat, Bcl-2 transduced primary CD4+ HIV latency, and central memory primary CD4+ T cells HIV latency models), differences in integration sites were found, especially when compared to in vivo samples. Further identification of in vivo integration site diversity using HC unlocks potential drug targets for the complete treatment of HIV (see also [91]). For example, HC was conducted on a disease-free HIV seropositive woman to investigate integration sites and reveal clues to her resistance to HIV without therapeutic intervention [92]. Based on the results, the authors suggested that her gut microbiota might be involved in the degradation of HIV genomes on integration via hyperactivating APOBEC3 enzymes, which may be an avenue for HIV treatment in the future. 

Determining integration sites in retroviruses is critical for developing therapeutic measures to combat infectious diseases. To determine integration sites for two retrovirus genomes—HIV-1 (161 baits) and HTLV-1 (148 baits)—baits were designed using a single reference for each [93]. However, given the high genomic variability of HIV types and subtypes, a more comprehensive bait panel was generated using genome alignments from HIV-1 and HIV-2 as references to recover sequences from clinical samples for genomic surveillance [94]. Indeed, up to 50 new HIV strains were characterised from clinical samples collected from Africa (five countries) and Thailand, contributing to surveillance and epidemiological studies. Separately, samples from the Democratic Republic of Congo were enriched using the same baits, leading to the formal establishment of subtype L in HIV-1 [95]. The different studies on the genetically variable HIV highlighted the versatility of HC in bait design—tailored panels can be designed depending on the research question. Identification of new strains of HIV through continuous surveillance efforts using HC methods aids in our understanding of the epidemiology of HIV, which could help in the mitigation and control of infections, particularly in regions rife with infections yet have limited access to resources [96]. 

#### 2.2.3. Unravelling Discrepancies in Genomes between Clinical and Cultured Samples in Herpesviruses

Traditionally, enrichment of pathogen sequences can be achieved via culturing. However, viral sequence discrepancies between passaged and clinical samples have been recorded. For example, RNA viruses are subject to exhibit high rates of mutations due to RNA-dependent RNA polymerases (RdRp), leading to single-nucleotide variants and insertions and deletions (indels), exemplified in influenza A (H3N2) [97,98], poliovirus, and dengue viruses (DENV) [99]. Nevertheless, DNA viruses are not exempt from this phenomenon, as observed in some herpesviruses. For example, using a novel panel designed on a single reference, a sizable deletion in one of the open reading frames (ORF) of the laboratory strain VZV32 (ORF 12: 2158 bp deletion) was found after propagating in fibroblast cells, despite being considered to be one of the most genetically stable herpesviruses [100]. Primarily, ORF12 is involved in Akt phosphorylation and the cell cycle; subsequent deletion in ORF12 could suggest that the proteins from ORF12 and ORF13 might have different roles in normal and cancerous cells [101]. 

Herpes simplex virus type 2 (HSV-2), a sexually transmitted pathogen, infects approximately 500 million people globally, whereas its counterpart, HSV type 1 (HSV-1), is much more prevalent in the general population, infecting some 3.5 billion people, but is usually transmitted via oral-to-oral contact instead [102]. Given that HSV-1 and HSV-2 genomes share only approximately 50% homology [103], a panel of baits designed exclusively for HSV-2 could only recover ~30% of HSV-1 genomes, which prompted a strategy to design baits for HSV-1 and HSV-2 separately [104]. Using a single HSV-1 and HSV-2 genome, the design comprised 1258 and 1285 baits (120 nt), respectively, each. Testing of the baits on both clinical and cultured samples revealed a lack of any significant difference in genome recovered. Therefore, culture-based techniques were recommended as viable options for enrichment and subsequent sequencing and genome recovery for HSV-1 and HSV-2 (see [105]). Regardless of genomic stability, we caution that regardless of DNA or RNA viruses, all herpesviruses cultured over multiple passages and time are likely subject to selection pressures [106]. Therefore, the decision on the method for genome recovery—HC or culture—has to be carefully considered and up to the discretion of the researcher and/or clinician.

#### 2.2.4. Broad-Range Viral Bait Panels and Clinical Metagenomics

Diagnosing infectious diseases using molecular techniques is routine—a physician conducts a differential diagnosis, orders a test (e.g., qPCR), and the results will either confirm or invalidate their hypothesis. However, in cases whereby the standard panel of tests fails to determine the causative agent of disease, clinical metagenomics (CM)—deep sequencing of a sample to recover pathogen sequences—may help to identify potential targets [3,46,107]. In a landmark study, infection by *Leptospira* was confirmed in a patient with meningitis by deep sequencing of a cerebrospinal fluid (CSF) sample [108] (see also [109]). However, due to overwhelming background noise from the host, enrichment of pathogen nucleic acids might be required, which can be achieved using HC. Furthermore, HC might be able to recover targets that qPCR was unable to: Mielonen et al. [110] were able to expand the repertoire of viruses targeted from FFPE samples to 38 using HC [111], beyond the 11 tested with qPCR. Beyond corroborating qPCR results, other viruses (i.e., MCPyV, BK polyomavirus (BKPyV), and JC polyomavirus (JCV)) were detected, and a false negative result for human betaherpesvirus 7 (HHV-7) was also corrected. Nevertheless, the more sensitive method of PCR has outperformed HC, such as if broad-range viral baits with lower sensitivity to certain taxa are used, which reduces capture and recovery [112]. 

Viruses are among the most genetically diverse clades, and their polyphyletic origin precludes conserved markers from being targeted. Therefore, an early panel, Virocap, used 337 viral species genomes as a reference for bait design [113]. The Virocap panel comprised approximately 2 million baits and was applied to a study detecting gastrointestinal (GI) viruses in transplant recipients for hematopoietic stem cells, specifically to differentiate between graft-versus-host disease or GI disease caused by viral pathogens [114]. Common culprits such as norovirus (see also [115]) and adenovirus positive in PCR diagnostics were also positive using HC, but a diversity of clinically relevant viruses, especially in those without any positive PCR result for common GI disease viral pathogens, were also captured, once again highlighting the need for judicious interpretation of results is required when using CM as a possible avenue for diagnosis. 

The advent of more efficient design algorithms resulted in a markedly smaller number of baits, in that of panels titled ViroFind [116] and V_ALL_ [117]. ViroFind was designed based on 535 viruses that infect humans or cause zoonosis (165,433 baits) and was used to characterise populations of viruses in five patients diagnosed with progressive multifocal leukoencephalopathy (PML) to be compared against 18 people with no known neurological disease. Enriching from clinical samples with PML (33- to 127-fold), they found complex populations of JCV with high genetic divergences and specific mutations in the viral capsid protein VP1 gene linked to tissue tropism, thereby increasing the fitness of the virus. V_ALL_, designed across 356 species of human pathogenic viruses for complete genome recovery, was implemented in a cohort of subjects (*n* = 25) with meningitis and encephalitis to augment metagenomic sequencing to capture the diversity of pathogens present. Notably, the pitfalls of relying solely on clinical metagenomics alone were highlighted in this study. While one subject with possible EBV infection (based on symptoms presented) had corresponding EBV reads recovered despite a negative PCR result, other subjects with the same HTS and PCR results did not have symptoms concordant with EBV infection, making it difficult to determine the contribution of EBV to their condition [118]. 

VirCapSeq-VERT was first introduced in 2015 as a panel comprising millions of baits designed using 342,438 coding sequences from viruses known to infect vertebrates as a reference [119]. Subsequently, it was updated and validated from clinical plasma and nasal samples [120]. To date, VirCapSeq-VERT has been tested in clinical metagenomic studies for respiratory diseases [120,121,122,123], neuroinfectious diseases [44,124], and autoimmune diseases [112]. VirCapSeq-VERT is a useful exploratory tool for identifying potential infectious agents when standard tests have failed to produce a conclusive diagnosis. To identify potential causative pathogens from febrile patients in Tanzania with unknown causes, plasma samples collected were tested using VirCapSeq-VERT [125]. However, recovery of multiple pathogens, such as DENV, West Nile virus (WNV), and human pegivirus (HPgV), stymied the team, preventing a conclusive diagnosis. Interestingly, in one sample, EBV sequences (*n* = 3) were recovered post-HC, which was corroborated using qPCR with a low C_T_ value, but no reads were recovered using unbiased sequencing. The recovery of sequences from multiple pathogens could indicate co-infections, but we echo the authors’ sentiment that HTS alone might not be indicative of a causal link, as results may be confounded by factors such as commensals or contamination (see [46,126]). Similarly, in cases of meningoencephalitis, the application of VirCapSeq-VERT managed to identify potential pathogens of interest, including unexpected ones [124], but results remained inconclusive and even failed to detect or identify alternative pathogens (i.e., HPgV) to those confirmed by clinical testing (i.e., VCV, JCV, and HSV-2) [44], albeit in a small sample size (*n* = 8). Finally, while the comprehensive viral research panel by Twist Bioscience, which targets over 3000+ viruses and is similar in principle to VirCapSeq-VERT, was used successfully for the detection of viruses implicated in patients suffering from meningoencephalitis, the negative PCR tests gave researchers pause. As a result, they, too, recommended careful interpretation of results by physicians under the advice of virologists and bioinformaticians [127]. 

Overall, CM hold great promise as a tool for pathogen identification, particularly for novel or indeterminate sources of infection. However, there are many challenges to overcome for it to be a proper diagnostic tool [4,46,107,126]. Currently, HC may complement CM by enriching for potential pathogens of interest; given the flexibility of bait design, multiple sets for different diseases can be designed to improve capture efficacy and reduce costs in the future. Furthermore, improvements to current baits and the availability of commercial options can complement the use cases of HC from complex samples. Critically, judicious curation of data and proper bioinformatic methods need to be implemented (e.g., removing host reads, accounting for cross-contamination of samples using negative controls, and accounting for commensals during interpretation of results). Interpretations should be aligned with the clinical presentation of the patients, reviewed by a physician. Until more research has been conducted across larger cohorts, with standardization of analytical techniques and interpretation of results corroborated by empirical samples, prudence in using CM with HC as a diagnostic tool should be exercised.

### 2.3. Parasites

#### 2.3.1. Malaria

*Plasmodium* spp. is a parasitic alveolate that causes malaria, with only five out of over 200 species known to infect humans [128]. Between 1955 and 1969, the World Health Organisation (WHO) launched the Global Malaria Eradication Programme (GMEP), and efforts at the eradication of this disease have continued to receive significant attention [129]. Despite the global movement, malaria continues to take an extreme toll on mankind, infecting millions around the world [130,131]. Among the five species that are known to infect humans, two are of particular interest: *P. falciparum* and *P. vivax*. In the early studies of employing HC, shearing of gDNA from cultured samples was used as baits for HC of the two parasite sequences [132,133,134]. Testing of the baits from the aforementioned studies demonstrated positive results, with an enrichment of up to 40-fold observed [132]. Interestingly, it was observed that AT-rich non-coding regions were less represented compared to GC-rich regions, a phenomenon particularly relevant to the AT-rich genome in *P. falciparum* (80.6%) [133]. More research is required into unbalanced nucleotide composition genomes, such as those being AT- or GC-rich. More recently, HC baits were designed based on *P. vivax* genomes to enrich for transcripts in order to characterise the transcriptomes of hypnozoites [135], laying the foundation for transcriptome characterization across the life cycle of the parasite, contributing towards the identification of potential drug and vaccine targets.

#### 2.3.2. Neglected Tropical Diseases (NTD)

Schistosomiasis is one of the top five neglected tropical diseases (NTD), posing a heavy burden on society, and global change wrought by anthropogenic activities is likely to exacerbate the risks and burdens of parasite infections in the near future [136,137]. Currently, baits are designed to primarily target exons in either *Schistosoma haematobium* or *S. masoni*, which is useful for identifying loci involved in drug resistance, candidate vaccines, and population genomics [138,139,140,141]. For example, the baits designed by Le Clec’h et al. [141] were used to determine an ancient introgression event of the invadolysin gene from *S. bovis* into *S. haematobium*, a gene of interest involved in infectious pathology within the host [142,143]. While they were unable to determine the functionality of the *S. bovis* invadolysin allele in *S. haematobium*, they recommended that future analyses be conducted on this gene. Genomic tools can elucidate epidemiology, control the spread of infection, identify pathogens, and contribute towards eliminating schistosomiasis, which can be augmented by HC [74].

Another NTD, leishmaniasis, affects millions across the world, causing tens of thousands of fatalities annually, but remains poorly studied [144,145,146]. Sequencing of *Leishmania* is complicated: they have either a flagellated (promastigote) or aflagellated form (amistagote) [147]. Genomes have been sequenced in both stages [148,149,150,151,152], and differences exist in genome architecture: aneuploidy in amastigotes cultivated from Syrian golden hamsters was lower compared to in vitro promastigotes [153]. Similarly, clinical samples of *L. donovani* also exhibited lower levels of aneuploidy, exemplified by sequencing genomes directly from leishmaniasis specimens in India via HC, using 218,904 (120 nt) baits spanning 26 Mbp of the 32 Mbp genome [152]. Multiple instances of polyclonal infections were identified, which is also characteristic of counterpart parasite *Plasmodium* [154]. Given the difference between in vitro and in vivo results, it might be advisable to obtain data directly from clinical samples instead for *Leishmania*, for both genomic and transcriptomic data, to better understand the in vivo response of the pathogen and thereby develop strategies to counter *Leishmania* infections. 

#### 2.3.3. Hybrid Capture and the Future of Neglected Tropical Diseases

Genomic studies of parasites have accelerated efforts in the management of parasitic infections through identifying potential targets for vaccine development [148,155,156], characterisation of drug-resistant loci [157,158], and elucidating evolutionary patterns and population genomics [159,160]. However, HC in parasites remains scarce, likely due to the lack of funding within the field, even for malaria, which ranks among the most well-known parasitic diseases [161]. Currently, many parasitic diseases are classified as “neglected tropical diseases (NTD)” by the World Health Organization (WHO), such as dracunculiasis, echinococcosis, and schistosomiasis. Unfortunately, NTDs are largely neglected because of economic and social imbalances—they disproportionately affect those with lower socioeconomic status [162,163]. Parasitic infections are emblematic of developing countries, stemming from limited access to clean water, food contamination, and other unhygienic practices, among other reasons [164,165,166]. While the need to tackle NTDs has been increasingly recognised, new challenges such as drug-resistant phenotypes, lack of vaccine candidates, and the lack of research funds threaten the efficacy of efforts underway [167,168]. While it is beyond the scope of this review to discuss the economic challenges that NTDs face, we highlight an article by Mohan et al. [146] that specifically extolls the economic benefits of vaccine development in leishmaniasis to manufacturers. 

From this review, we found only 10 studies to date on parasitic pathogens in humans using HC (Figure 1), which are limited to a handful of diseases. With millions of new infections annually, it is imperative for the global public health movement to invest more resources into combating parasitic infections, especially with rapid urbanization and increasing incidences of parasitic infections on a global scale [169]. The space of HC in the realm of parasites remains largely unexplored, and the impact of research into this field may have far and wide-reaching benefits to mankind. For example, genome sequencing of cultivated *Trypanosoma cruzi* identified loci correlated with drug-resistance [170]; HC can reduce costs associated with whole-genome/transcriptome sequencing from cultured samples, thereby alleviating the economic burden on researchers working on NTDs. Eventually, an improved understanding of NTDs can help efforts in management and subsequent eradication, which is especially important for developing countries with populations of lower socioeconomic status.

### 2.4. Fungi

The number of studies employing HC in human fungal pathogens is extremely limited: we were only able to find three studies, all related to *Candida* spp. transcriptomics for gene expression analyses (Figure 1; Table S1). In the earliest study, researchers targeted the transcriptome of *C. albicans* using 55,342 baits designed across 6,094 open reading frames (ORFs). Testing on both in vitro and in vivo (*Mus musculus* and *Galleria mellonella*) samples obtained up to a 1,600-fold increase [171]. A similar strategy was adopted for *C. glabrata*, except two sets of baits were designed: one comprising 49,789 baits designed across 4995 ORFs (cell wall adhesins excluded), and another with 49,964 baits across 5134 ORFs (cell wall adhesins included) [172]. The baits were able to enrich fungal transcripts recovery between 750- and 970-fold; subsequent gene expression analyses of urinary tract infections in mice led to the identification of four new virulence genes that could be potential targets for treatment. Finally, Hovhannisyan et al. [173] embarked on an ambitious strategy, designing baits for four phylogenetically diverse *Candida* species (*C. albicans*, *C. glabrata*, *C. parapsilosis*, and *C. tropicalis*). Capitalising on the previous two studies, they extended bait design beyond ORFs only and included annotated features from non-coding regions. Furthermore, the experiment was conducted on spiked human vaginal swabs instead of murine or insect models, with a 1.4- to 17-fold enrichment, and they demonstrated that genotyping of captured sequences allows for the identification of strain and potentially antifungal susceptibility profiling. 

To the best of our knowledge, no study has attempted to recover fungal sequences from clinical samples using HC. This could be due to the difficulty in bait design for fungal pathogens—fungi have relatively large genomes [174], which would be costly for bait design in contrast to their bacterial and viral counterparts with considerably smaller genomes in general. Furthermore, of the 607 fungi infecting humans as retrieved from a curated list, only 353 have genome sequences available [175]. While it might be sufficient for a comprehensive HC panel to be deisgned, distant clades might fare poorly post-HC. There is a need for more genomes to be sequenced and assembled, but we recognize that it is a costly and difficult undertaking.With over a million deaths per year being fungal-related [176], more attention should be paid to the knowledge, treatment, and control of fungal pathogens [177,178], which can be augmented by judicious bait design and HC techniques (see also Section 5.1 Bait Design).

## 3. Biosurveillance and Emerging Viral Infectious Diseases

More than half of human pathogens stem from zoonotic sources; zoonoses were thrown into the spotlight because of the COVID-19 pandemic [179,180]. In the last decade alone, the emergence and re-emergence of viral infections were reported globally [181,182,183], and future epidemics are inevitable [184,185]. Climate change, biodiversity loss, and population mobility today are likely to exacerbate the effects of zoonoses [185,186]. Technological advances in genomics have transformed the playing field, allowing for surveillance and early detection in real-time [184,187,188,189], which can be augmented using HC. Coupled with lessons learned from the recent pandemic (e.g., global collaborations and surveillance for pathogens that might result in a pandemic), the destructive force of the next deadly global pathogen can be mitigated considerably [190]. Furthermore, by identifying and studying pathogens with pandemic potentials via a representative taxon (e.g., SARS-CoV-1 as a model to understand SARS-CoV-2), responses to the pathogen can be implemented rapidly and effectively (e.g., healthcare, vaccine development). 

### 3.1. Arthropod-Borne Viruses

#### 3.1.1. Mosquito-Borne Viruses 

Mosquito-borne diseases (MBD) are a bane to public healthcare around the world, costing billions of dollars each year [191]. Biosurveillance and genomic epidemiology are crucial in shedding light on the biology, diversity, and transmission of MBD, which can be achieved using HC. Conducting environmental surveillance of mosquitoes from the United States using V_ALL_ for human pathogens, researchers were able to detect two pathogens of interest: WNV and Culex flavivirus (CxFV) [117]. Whether West Nile infection is an emerging or neglected disease is up for contention [192], but experts agree that monitoring of WNV is critical in management and control strategies [193,194], which can be implemented using HC. 

Using *Aedes* mosquitoes as a vector to infect primates, Zika virus (ZIKV) infections typically present mild symptoms, although some neurological conditions such as microencephaly and meningoencephalitis have been associated with infections. Due to the rapid spread of ZIKV in the Americas, extensive research has been conducted into the genomic epidemiology of the virus. Shortly after the beginning of the ZIKV epidemic in 2015, Naccache et al. [195] used HC to recover genomes from patients in Brazil and found evidence for a Bahia-specific lineage that has been circulating since 2014. Subsequently, to reconstruct the introduction and transmission chains of ZIKV in Miami, Grubaugh et al. [196] designed their own baits (866 baits, 80 nt) for the enrichment and assembly of ZIKV genomes not only from patients but also from *A. aegypti* mosquitoes for biosurveillance. Using genomic data, the authors were able to trace introductions to Miami from the Caribbean multiple times and further ascertain that local transmission has occurred. Moving to the south, Thézé et al. [197] embarked on a similar study in Central America and Mexico (CAM), using baits designed across 44 ZIKV genomes to supplement sequences obtained from metagenomic sequencing. They found that despite multiple introductions of ZIKV into CAM, only one lineage introduced became established (clade B sensu [197]), which likely occurred in Honduras in the summer of 2014. The seasonal change coincided with optimal conditions for the proliferation of its mosquito vector, accentuating the correlation between environmental conditions and MBD. Subsequently, this lineage was able to disperse into Guatemala, southern Mexico, and Nicaragua before being detected in November 2015. The introduction of this lineage likely occurred in Brazil, which was the origin of the introduction of ZIKV into the Americas. Detailed genomic epidemiology is especially useful for the management of diseases, as targeted approaches to controlling either the vector populations or cross-border movement can be implemented if necessary [198].

On a finer geographical scale, researchers were able to recover almost complete genomes from patients infected with ZIKV in Singapore in the 2016 outbreak, using baits designed to target three MBD viruses (ZIKV, DENV, and chikungunya virus (CHIKV)) from their novel algorithm, Baitmaker [199]. With the vector *Aedes* mosquitoes being endemic and common in Singapore, cases of ZIKV are not unheard of, albeit limited, in the island city-state. The state’s National Environmental Agency (NEA) continually surveys mosquitoes and wastewater in areas with active cases and proceeds with necessary vector control measures when there is reason to suspect an active viral population that has the potential to become a hotspot for infections. The combination of HC and environmental surveillance can guide early intervention measures, which aids in controlling the spread of diseases, thereby safeguarding public health interests. 

#### 3.1.2. Tick-Borne Viruses

Pathogens are also commonly transmitted via other arthropod vectors, such as ticks. Tickborne disease (TBD) comprises a diversity of pathogens—bacteria, viruses, and parasites—and is an emerging problem [200,201,202]. To detect TBD pathogens in the USA, researchers designed a panel of baits (TBDCapSeq) targeting 11 prevalent targets: nine bacteria and two virus species [203]. Testing of the baits directly on field-collected ticks, complete genomes for Powassan virus (POWV) from two ticks were recovered, highlighting the capability of HC in biosurveillance and monitoring of pathogen reservoirs. Crucially, POWV is a re-emerging TBD that causes meningoencephalitis, making it a model target for HC and biosurveillance [204]. Subsequently, TBDCapSeq was updated by adding one bacterium (*Francisella tularensis*) and two viruses (Colorado tick fever virus (CTFV) and Bourbon virus (BRBV)) [205]. Currently, TBD is typically not routinely included in the standard CSF PCR, but HC can be used to complement the identification of diseases. For instance, the use of broad-range V_ALL_ on clinical samples corroborated Powassan virus infection in two out of three serologically confirmed cases [118]. However, as previously discussed, HC and PCR results may differ and give conflicting results. Therefore, tried and tested diagnostic techniques must take precedence over CM techniques, and CM remains an auxiliary tool for diagnosticians to tap into.

### 3.2. Mammalian Zoonotic Viruses

#### 3.2.1. Lassa and Ebola Viruses

First documented in 1969, Lassa fever is caused by the Lassa virus (LASV), transmitted by the multimammate rat *Mastomys natalensis*, and remains a threat in West Africa [206,207]. Illustrating the difficulties of obtaining pathogen nucleic acids from clinical samples, direct sequencing of clinical and biological samples yielded poor recovery LASV material, despite extensive depletion of carrier and host RNA, albeit sufficient recovery of Ebola virus (EBOV) sequences was observed [208]. Therefore, to improve the recovery of LASV, two panels of baits were designed separately to account for genetic divergences between Nigeria and Sierra Leone clades. Following enrichment (average of 86-fold), the team successfully called intra-host single nucleotide variants (iSNVs) for genotyping of LASV from clinical samples. Empirical testing was also conducted on Lassa fever clinical samples collected from outbreaks in 2018 in Nigeria, using a broad-range panel V_ALL_ [117]. Despite the taxonomic breadth targeted by V_ALL_, fragments of LASV genomes for 22 out of 23 samples were recovered. In contrast, metagenomic sequencing without HC recovered sequences from only a single sample (see [209]). 

Before COVID-19, filovirus disease (FVD) was on WHO’s radar as a potential pandemic, particularly Ebola virus disease (EVD). Currently, EVD is thought to be an emerging infectious disease originating from a zoonotic source from a currently unknown animal reservoir in Central Africa, with bats being the most likely suspect (reviewed in [210]). Evidence for long-term persistence and eventual sexual transmission of EBOV was found in a HC study from a semen sample collected 179 days after EVD, albeit no sequences were recovered from metagenomic sequencing alone [211]. This provided valuable insights into the infectivity of EBOV over time, aiding in the management and control of the disease. In sum, it appears that with respect to clinical samples with RNA viruses, due to small genome sizes and overwhelming interference from host RNA, extensive depletion of background RNA, coupled with HC, would be a strategic approach to improve recovery of sequences from pathogens of interest.

#### 3.2.2. Coronaviruses

Since the SARS pandemic in 2003, much attention has been paid to the surveillance and identification of coronaviruses (CoV). Bats and pangolins are known reservoirs of CoVs [212,213]. Recent analyses surveyed SARS-CoV-2-related coronaviruses (SC2r-CoVs) diversity in pangolins via metagenomic sequencing [212,214]. Interrogating the diversity of SC2r-CoVs from 163 confiscated pangolin individuals smuggled into southwestern China, Peng et al. [215] designed baits on 50 SARS-CoV-2 genomes (502 baits) for enrichment of SC2r-CoV sequences. Notably, SC2r-CoVs in pangolins demonstrated high genetic diversity and could potentially be a source of zoonotic infectious diseases. Alarmingly, human pathogenic viruses were also directly identified from pangolins using metagenomic sequencing [216]. Their mammalian chiropteran counterparts are also notorious as reservoirs of CoVs, including pathogens such as severe acute respiratory syndrome coronavirus (SARS-CoV)-like and Middle East respiratory syndrome coronavirus (MERS-CoV)-like viruses that jumped to humans via intermediate hosts—civets and dromedaries respectively [217]. Bearing testament to concerns regarding coronaviruses, Lim et al. [218] designed 4303 baits (120 nt) for alpha- and betacoronaviruses across 90 genomes and was able to recover and assemble a novel *Betacoronavirus* BtCoV92 from *Cynopterus brachyotis* samples collected in Singapore. *Betacoronavirus* is the genus to which several deadly coronaviruses belong, making it especially pertinent for investigation. Subsequently, the same methodology on rectal swabs collected for CoV surveillance projects in China discovered nine new CoV genomes [219]. Similarly, Rousettus bat coronavirus GCCDC1 (RoBat-CoV GCCDC1) was also detected from samples collected in Singapore [220], using baits designed for filoviruses, respiratory viruses, and other viruses of interest with respect to biodefence and biosurveillance [221]. RoBat-CoV GCCDC1 is a coronavirus that was previously identified only from bats in Yúnnan, China, and the presence of Singapore samples expands the geographic range of the coronavirus, and monitoring can help in tracking bat coronavirus diversity and distribution. 

Out of Asia, Kuchinski et al. [222] designed baits against all bat CoV sequences (18,365 baits) for recovery of CoVs from bat swabs in the Democratic Republic of the Congo. An additional 1635 baits were designed from conserved motifs in non-bat CoVs to round the number of baits up to 20,000. Due to low nucleic acid concentrations, the authors were only able to recover < 93% of the genomes for five new CoVs solely via HC and had to augment their data with deep metagenomic sequencing to complete the genome assembly. Indeed, despite the positive results obtained from HC in general, ultradeep metagenomic sequencing might still be needed for the recovery of complete genomes due to the inability of HC baits to capture certain highly variable regions. Finally, researchers used VirCapSeq-VERT to successfully recover SARS-CoV-2 genomes from both clinical and environmental samples, supporting the notion that broad-range bait sets are able to contribute meaningfully to unknown or novel pathogens [123,223,224,225] (see also Section 4. Case Study: SARS-CoV-2).

#### 3.2.3. Monkeypox Virus

In the middle of 2022, monkeypox viral infections (mpox) surfaced on the public health radar right after the waning of COVID-19, only a few months after initial reports of a rapid rise in mpox infections concentrated in Europe, the WHO declared mpox to be a Public Health Emergency of International Concern. The potential for mpox as an emerging infectious disease has been raised several decades ago [226,227]. Monkeypox virus (MPXV) genomes in wild chimpanzee populations from Côte d’Ivoire using non-invasive sampling methods were investigated prior to the outbreak and collected over several decades [228]. Specifically, environmental samples (urine, faeces, fruit wedges, and flies) and tissue from carcasses were collected for the sampling of MPXV using baits designed by Arbour Biosciences for orthopoxviruses. Two lineages of MPXV in Taï National Park chimpanzees were discovered, and the results were able to trace the epidemiology of MPXV outbreaks in chimpanzees. Eastward in the Central African Republic (CAR), a panel for MPXV was designed using 10 complete genomes for testing on clinical samples collected from outbreaks between 2001 and 2018 [229]. Strikingly, molecular dating analyses revealed three distinct lineages that diverged in periods of political instability and migration, with widespread poverty. The authors postulated that the demand for bushmeat to alleviate living conditions facilitated zoonotic transmission of MPXV. Incursions into natural habitats in the region will only exacerbate the spread of mpox, with more human-wildlife interactions expected. 

Enrichment of MPXV sequences using HC has also been used to investigate outbreaks. In 2017, mpox reemerged in Nigeria, and baits from V_ALL_ were used to enrich MPXV sequences (from a maximum of 30 reads based on unbiased sequencing to a maximum of 20,000 mapped reads) [209]. Post-enrichment, the researchers were even able to verify the clade of MPXV (i.e., clade IIb), which was congruous with previous reports. Over in the West, baits were designed using MPXV 2022/MA001 strain to investigate contemporary outbreaks of mpox infections in the USA by enriching MPXV sequences from clinical samples [230]. Recovering identical genomes between people, researchers were able to trace ongoing community transmissions, but the low genetic diversity of MPXV hampered contact tracing efforts via whole-genome sequencing. The diversity of panels used to test for MPXV highlights the flexibility of HC for different purposes, arming researchers with the best tool for their investigations. 

#### 3.2.4. Moving Forward: Biosurveillance of Mammals

Considering that there have been two pandemics due to SARS-CoV in the 21st century alone and the diversity of novel coronaviruses identified by the different studies, the importance of continual environmental surveillance and preparedness for any new public health threats cannot be overstated. Indeed, mammal-borne zoonoses as the source of the next pandemic loom large over public health [231]. As we continue to investigate the origin and source of SARS-CoV-2, we need to increase the sampling and diversity of coronaviruses and beyond in our databases to better trace the evolutionary origins of not only SARS-CoV-2 but potentially, the next candidate for a global pandemic [232]. Only with more genomic sequences by sampling across a large cohort of mammals can we better prepare ourselves by implementing intervention measures (e.g., safe handling of animals and reduced wild-life human interactions) where possible. 

## 4. Case Study: SARS-CoV-2

**Figure 2:**
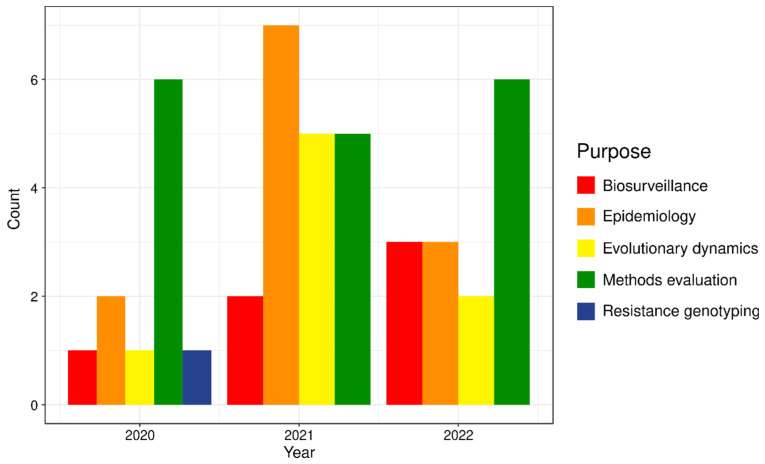
Number of studies (by year) investigating different aspects of SARS-CoV-2 using hybrid-capture target enrichment for sequence recovery between 2020 and 2022.

In December 2019, Wuhan, China, saw a sudden outbreak of pneumonia with no known causes. Subsequently, HTS of clinical samples revealed the culprit to be a novel betacoronavirus, which was eventually named SARS-CoV-2 [233,234]. The early epidemiology of SARS-CoV-2 was traced by Chowdhury and Oommen [235]: evidence for the global spread of the virus was confirmed by cases in Thailand, the USA, Japan, and India in January 2020. By March 2020, the WHO declared the COVID-19 pandemic, throwing the world into disarray. The introduction of nonpharmaceutical interventions (NPIs), such as social distancing and mandatory facemasks, together with the rapid development of vaccines, helped mitigate the devastation of COVID-19 [236]. Surveillance and contract tracing studies helped epidemiologists understand the transmission of the virus, including genomic surveillance and epidemiology, some of which were conducted using HC. Early studies on HC in SARS-CoV-2 in 2020 primarily verified the utility of HC by comparing across different methods for pathogen genome recovery (e.g., PCR, HC, and metagenomics), and the successful recovery of pathogen genomes led to subsequent studies on genomic epidemiology and evolution (Figure 2).

The genome sequence of SARS-CoV-2 isolate Wuhan-Hu-1 was made publicly available in mid-Jan 2020 [237], and studies adopting commercial kits for HC in SARS-CoV-2 were published mere months after [238,239]. Given the diversity of commercial panels available (Table S2), Rehn et al. [240] compared five different panels of efficacy. Expectedly, they found that taxon-specific panels produced the best results compared to broader-range panels (e.g., respiratory viruses panel). Beyond the choice of panels, studies also investigated and verified the efficacy of HC for SARS-CoV-2 sequences across bioinformatic pipelines [241], sequencing platforms [242,243], and even library preparation methods [244]. Generally, molecular techniques (e.g., metagenomic sequencing, HC, and PCR) performed comparably, but in SARS-CoV-2, as the pathogen has a relatively small genome (~30 kb), enrichment of sequences can be executed using dedicated primers for the entire genome (e.g., ARCTIC 4.1). However, due to the rapid mutation rate of the virus, amplicon dropouts may lead to the inability to determine lineages [245,246]. Furthermore, PCR may be susceptible to technical artefacts such as primer bias, which has led to contentious results [247]. To circumvent the drawbacks of metagenomics (i.e., cost) and PCR (i.e., primer specificity and technical artefacts), HC is a strong alternative (see [248]).

The recovery of SARS-CoV-2 genomes opened the doors for genomic epidemiology, biosurveillance, and phylogeny reconstructions. As infections raged on, multiple variants of concern (VOCs) appeared—e.g., Alpha, Beta, Gamma, Delta, and Omicron—a result of the remarkable capacity of SARS-CoV-2 for mutation, with variant recombination spawning multiple lineages within the VOCs [249]. Genome surveillance has also enabled strain diversity characterisation around different parts of the world, and divergence dating provides estimates of cladogenesis for different variants [250,251]. Different VOCs have unique clinical and biological characteristics, including pathogenicity; it is important to identify circulating and prevalent VOCs in order to implement appropriate controls [252]. Sequencing of 74,738 HC samples from the United States found that between January 2021 and September 2021, the SARS-CoV-2 Delta variant had effectively dominated almost every case of COVID-19 by September, after first being detected in March 2021 (>99.9%) [253]. Since the Delta variant had a much higher fatality compared to its predecessor, Alpha, more severe NPIs were advised to accommodate the rise in cases [254]. Relatedly, Zhang et al. [255] were able to use SARS-CoV-2 genomes recovered via HC and subsequent phylogeny reconstructions to find evidence of transmission and trace the spread of the virus in asymptomatic patients, which is often overlooked in models calculating the reproductive number (R_0_) but crucial for understanding the spread of the disease [256,257]. Indeed, HC was extensively employed in tracing outbreak patterns during COVID-19 (Figure 2; Table S1), enhancing our understanding of the pandemic’s evolutionary dynamics, virulence, and management.

Environmental samples can be tested for SARS-CoV-2 and other viruses using HC, enabling surveillance and guiding policy decisions. Coronaviruses, including SARS-CoV-2, have been successfully extracted from sewage/wastewater using HC, which were generally concordant with clinical specimens collected from the same region [224,258]. Non-invasive sampling can be readily adapted to provide insight into the circulating strains, and consistent surveys using broad-range bait panels enhance biosurveillance efforts and discovery of new pathogens [209,223,224], which can be used to guide policy decisions [123,259]. Beyond sewage samples, researchers in Kuwait sought to characterise respiratory viruses from hospital air using a commercial panel [260]. Nosocomial transmission of respiratory pathogens remains a threat to recovering patients [261], and they were able to identify SARS-CoV-2 from hospital air, together with other respiratory pathogens of interest such as human adenoviruses, respiratory syncytial virus, and influenza B. In hospital settings, HC can be employed for the identification and genotyping of pathogens for genomic epidemiology, to reduce the spread of nosocomial infecitons [262].. From clinical to environmental samples, HC proved to be a useful and versatile technique during the recent pandemic, cementing its value in the toolbox of molecular biologists.

## 5. The Future of Hybrid Capture

### 5.1. Bait Design

Bait design can be a tricky process, with the complexities increasing through deep time and genome sizes. The early forays into HC primarily relied on a tiling method for bait design, but there has been an increasing number of algorithms available for bait design in recent years to accommodate complex panel design. New methods primarily focused on human pathogens include CATCH [117], BaitMaker [199], HUBDesign [45], MrBait [263], ProbeTools [264], and Syotti [265]. Alternative options popular in evolutionary biology include phyluce [266], BaitFisher [267], BaitTools [268], and supeRbaits [269]. With the diversity of algorithms available, researchers need to carefully consider the pros and cons of each programme and decide on the most suitable method for bait design for their target(s) of interest.

The availability of options allows for flexibility in bait design, depending on the research question. For example, HUBDesign would be suitable for designing a panel spanning a broad taxonomic coverage, as baits are designed by clustering samples based on an evolutionary framework and conserved, shared genes, thereby reducing the number of baits required. In contrast, programs such as ProbeTools or Syotti might be a better choice for polyphyletic viruses, as they operate on a k-mer and Hamming distance approach for comprehensive capture of targeted regions. However, Syotti does not have a built-in bait filtration function and post-processing of baits; parameters to consider include GC content (between 40 and 65%), masking baits at repetitive regions (or prior to bait design), and ensuring baits do not self-hybridise to each other should be conducted post-design.

Finally, for fungi and parasites with larger genomes, bait design can be a tricky process. The majority of studies discussed in this review for the two taxa targeted the enrichment of transcripts for characterization of transcriptomes or for a small number of taxa, which can be easily achieved using a tiling design. However, programs such as phyluce and BaitFisher may be useful for designing a broad-range panel to capture sequences for subsequent pathogen identification. The former extracts ultra-conserved elements from reference genomes or transcriptomes, whereas the latter is design baits based on orthologous genes identified from transcriptomes, with the option of alignment cutting to account for exon-intron boundaries in parasites and fungi. These properties make the programs suitable for capturing shared, conserved loci across deep evolutionary divergences. The results can then be used for either classification (e.g., [270,271]) or phylogeny reconstructions to identify current or novel pathogens. 

### 5.2. Long-Read Sequencing

Capture of target sequences for HC was previously designed for short-read sequencing platforms, such as Illumina or Ion Torrent [18,53,242]. With the advent of long-read sequencing, such as PacBio or Oxford Nanopore Technologies (ONT), some recent studies have combined HC and long-read sequencing [38,242,243,272], albeit being few and far between. Long reads are suitable for accurate assemblies, which have downstream analytical implications such as comparative genomics and pathogen identification [273,274]. In a response letter to reviewers by Kuchinski et al. [222], they found several limitations of MinION sequencing in conjunction with HC. Most importantly, they only managed to recover sequences comparable in length to those from Illumina libraries, defeating the purpose of using long-read sequencers. Similarly, during the recent pandemic, a HC protocol developed by PacBio had a recommended insert size of only 675 bp to cover SARS-CoV-2 sequences, which is only marginally longer than the standard insert size in Illumina sequencing [243,275]. 

Despite the challenges in HC for long-read sequencing, Slizvoskiy et al. [38] managed to characterise AMR genes from mock and stool samples using a custom panel designed with a 1× tiling (120 bp baits) with PacBio sequencing. Using MinION sequencing, Eckert et al. [272] recovered a mean read length of between ~1000 and 3000 bp for HCMV and *M. tuberculosis*, albeit with an insert size of >10 kb. Currently, more research is required into suitable bait design techniques—numerous overlapping baits for specific loci may compromise the integrity of long fragments due to mechanical stress, and other factors such as bait specificity and PCR biases for shorter fragments will be important considerations. Specific algorithms tailored for HC and long-read sequencing may be required, coupled with empirical tests to optimise methods. Ultimately, we remain excited about the prospects for combining HC with long-read sequencing technologies in the field of human pathogen genomics. 

## 6. Conclusions

In this review, we demonstrate that HC methods are especially useful for the enrichment of human pathogen sequences as they can be designed for disease across multiple scales of divergence, loci, or other desired configurations. Improvements in bait design algorithms and an increased interest in HC will likely result in a diversity of panels enriching human pathogens from different types of samples (e.g., environmental and clinical). Indeed, HC is a viable and useful tool for the enrichment of sequences at low concentrations, especially when capturing across a broader taxonomic range. Pathogen genomic sequences can be used for pathogen identification, genomic epidemiology, and environmental surveillance, making it invaluable in the study of outbreaks. In addition, the applications of HC can extend to transcriptome characterisation and determining differential gene expression, furthering our standing of host–pathogen interactions. Future research into the combination of long-read sequencing and HC will produce more robust results, especially in difficult-to-sequence/assemble regions, and we suspect novel applications of HC will likely arise as the field matures.

## Data Availability

The data used to prepare the figures are available in the Supplementary Materials.

## Supplementary Materials

The following supporting information can be downloaded at https://www.mdpi.com/article/10.3390/pathogens13040275/s1, Table S1: List of studies employing hybrid capture for human pathogens.; Table S2: List of commercial kits for hybrid capture target enrichment of human pathogens.
